# The effect of mindfulness training prior to total joint arthroplasty on post-operative pain and physical function: study protocol for a randomised controlled trial

**DOI:** 10.1186/1745-6215-15-208

**Published:** 2014-06-05

**Authors:** Michelle M Dowsey, David J Castle, Simon R Knowles, Kaveh Monshat, Michael R Salzberg, Peter F M Choong

**Affiliations:** 1 The University of Melbourne Department of Surgery, St. Vincent's Hospital Melbourne, 35 Victoria parade, Fitzroy 3065, Victoria, Australia; 2 Department of Psychiatry, St Vincent’s Mental Health, The University of Melbourne, 41 Victoria Parade, Fitzroy, Victoria 3065, Australia; 3 Faculty of Life and Social Sciences, Swinburne University of Technology, Burwood Road, Hawthorn, Victoria 3122, Australia

**Keywords:** Function, Mindfulness, Outcomes, Pain, Total joint arthroplasty

## Abstract

**Background:**

Osteoarthritis is a leading cause of disability in developed nations. In Australia it afflicts 16.5% of the adult population. Total joint arthroplasty is considered the treatment of choice for end stage osteoarthritis. The number of total joint arthroplasties undertaken in Australia has doubled over the last decade (more than 80,000 procedures in 2011). The incidence of pre-operative psychological distress in this group of patients is reported between 30% and 60% and pre-operative psychological distress is associated with poorer pain and functional outcomes after surgery. This study will use a mindfulness-based psychological intervention to enhance outcomes in people undergoing total joint arthroplasty and, in addition, will test hypotheses about coping with chronic illness in an aged population. This study is the first of its kind and will provide a greater understanding of the role of a mental health enhancement program on the physical recovery of total joint arthroplasty patients.

**Methods/Design:**

One hundred and fifty people with end-stage arthritis on the waiting list for total hip or knee arthroplasty will be recruited and randomly allocated to one of two groups using computer-generated block randomisation. A randomised controlled trial adhering to CONSORT guidelines will evaluate the efficacy of a mindfulness training program (weekly group-based classes in mindfulness practice, 2 ½ hours, for 8 weeks plus a 7-hour Saturday session in Week 6) prior to total joint arthroplasty, compared to a “standard care” group who will undergo routine total joint arthroplasty. Primary outcomes will be evaluated by a blinded examiner at baseline, 3 and 12 months post-surgery, using a validated self-reported pain and physical function scale. Secondary outcomes will include i) a range of validated measures of psychological wellbeing and ii) health economic analysis. All analyses will be conducted on an intention to treat basis using linear regression models. Health economic modelling will be applied to estimate the potential cost-effectiveness of mindfulness training and total joint arthroplasty.

**Trial registration:**

Australian New Zealand Clinical Trials Registry (ANZCTRN12611001184965). Date of registration; 15th November 2011.

## Background

Osteoarthritis is a leading cause of disability in developed nations. In Australia, it afflicts 16.5% of the adult population and imposes a significant economic burden [1]. Total joint arthroplasty (TJA) is considered the treatment of choice for end-stage osteoarthritis. However, the success of TJA, a high volume, expensive procedure, is undermined by pre-operative psychological distress, a known predictor of suboptimal improvement after surgery. Psychological co-morbidities and traits reported in TJA patients include depression, anxiety, neuroticism, catastrophizing, poor self-esteem, and low self-efficacy, all of which are associated with poorer function and greater pain after TJA in short and longer term follow-up studies [2-7].

Our recent comprehensive literature review found pre-operative psychological distress to be an independent predictor of pain and function after TJA in a majority of published studies [8]. This relationship has also been noted for other major surgical procedures, e.g., herniated disc surgery [9] and coronary artery bypass graft surgery [10]. In a large prospective study involving more than 1,000 TJA recipients, we showed that poorer pre-operative mental health scores were associated with weight gain after surgery [11]. Obesity is over-represented in TJA candidates (hip patients > 40% obese, knee > 60%) and only 10% show clinically significant weight loss after surgery [11,12]; obesity itself is strongly associated with depression [13]. Pre-operative psychological distress is also associated with excessive analgesic intake, higher rates of hospital readmission, and long-erm mortality [14].

Despite such findings, there is a dearth of literature examining psychological interventions or programs in surgical patients generally, and, to the best of our knowledge, there are no published intervention studies specifically for TJA. Thus, there is a pressing need to examine whether implementing a mental health enhancement program improves postoperative outcomes in patients undergoing TJA, a question with relevance to other surgical programs and to the health of the aged more generally.

Mindfulness training (MT) is derived mainly from Eastern-based philosophical traditions, with mindfulness-based concepts and therapies widely incorporated into experimental and clinical psychology and psychiatry in the past 30 years [15]. Although there is a spectrum of mindfulness-related concepts, we adopt the definition of Kabat-Zinn, where mindfulness is “*the awareness that emerges through paying attention on purpose, in the present moment, and non-judgmentally to the unfolding of experience moment by moment*” [16] (p. 145). This is the basis of an approach known as “mindfulness-based stress reduction” (which we will here refer to as MT for the sake of brevity), the approach employed in most studies of physical health conditions [15].

Benefits of MT have been demonstrated for psychiatric disorders [15,17] and in stressed healthy cohorts [18], but of most relevance to this proposal is its use in older adults [19] and in patients with physically debilitating conditions such as multiple sclerosis [20], fibromyalgia [21], arthritis [22], and chronic pain [23]. Randomised controlled trials in pain patients show efficacy for psychological distress (in general and for symptoms of depression and anxiety in particular), for health related quality of life, as well as for fatigue, pain, functioning, and somatic complaints. Most studies show low attrition (15%) [24] and sustained improvements (particularly in functioning) at up to 3 years [15]. Most psychological interventions for physically ill subjects have arisen out of psychotherapies for the mentally ill (notably cognitive behaviour therapy). In contrast, MT is useful in distressed patients who do not necessarily meet diagnostic criteria for mental illness [25].

Keeping in mind the absence of previously established psychological interventions in arthroplasty, we propose that MT prior to TJA is likely to be a particularly suitable and effective intervention, leading to better outcomes. The primary aim of this research is to determine if psychological well-being and physical recovery are improved in TJA patients if surgery is preceded by a mental health enhancement program. Two exploratory aims of this study will be: i) to compare the cost effectiveness of this combination versus standard of care; ii) to investigate the influence of mindfulness training on candidate mediators of health outcomes drawn from psychological research on adaptation to illness–notably cognitive distortions, illness beliefs, and coping styles.

## Methods

### Experimental design

A randomised controlled trial of individuals undergoing TJA for end-stage arthritis. Patients in the intervention arm will undergo mindfulness training prior to TJA. Patients in the control arm will undergo TJA only. Outcomes will be compared at 12 months after TJA. The study strategy will be registered, constructed, and presented according to the recommendations of the CONSORT statement [26].

### Participants

Participants on the TJA waiting list at St. Vincent’s Hospital Melbourne (SVHM) who present for pre-operative assessment with moderate to severe psychological distress will be invited to participate in the study. Pre-operative assessment is conducted in the orthopaedic pre-admission clinic at SVHM at which point physical and psychological well-being scores are routinely captured using the Short Form-12 (SF-12) questionnaire [27]. Attendance at the orthopaedic pre-admission clinic is mandatory prior to TJA. Here, patients undergo health assessment and education and provide informed consent to TJA. The team comprises an orthopaedic surgeon, resident medical officer, and nursing and allied health staff. After initial screening, potentially suitable patients will be given a plain English statement detailing the nature of the study and the commitment required. For patients willing to proceed, informed consent for the study will be obtained by the research associate.

### Inclusion

i) On the surgical waiting list for primary TJA for end-stage osteoarthritis at SVHM.

ii) Presents with moderate to severe pre-operative psychological distress (SF-12 MCS < 40). The SF-12 measures physical and psychological well-being and is validated for use in the clinical setting and Australian population. A score of less than 40 on the mental component summary indicates moderate psychological distress [27].

### Exclusion

i) Revision surgery or surgery for neoplastic disease.

ii) Inability to provide informed consent due to mental incompetence (e.g., intellectual disability, dementia; mild cognitive impairment would not be cause for exclusion).

iii) Active drug or alcohol use disorder which in the opinion of the investigators makes the patient unsuitable for participation in the trial.

iv) Limited English language fluency.

v) Evidence at interview or on the Memory Complaints Questionnaire [28] of presumptive cognitive impairment requiring clinical assessment.

### Intervention

The intervention group will undergo MT prior to TJA, with a ‘booster’ session at 3 months post-surgery. The 8-week program will be conducted on-site through the Department of Psychiatry at SVHM by MBSR-qualified investigators and will be repeated every 10 weeks during the recruitment phase. The program consists of: i) a personal interview to establish rapport, undertake a brief psychiatric assessment and arrive at realistic goals; ii) weekly group-based classes in mindfulness practice, 2 ½ hours, 10–20 participants, for 8 weeks plus a 7-hour Saturday session in Week 6. MT is manualized with each class covering specific, predetermined MT exercises and topics. Yoga based exercises are adapted to be performed in either sitting or lying, to accommodate the potential mobility limitations of the study population; iii) home-based assignments to encourage regular practice of learned skills, utilising a workbook and CDs with recorded meditation instructions; iv) a post-intervention evaluation interview of personal experiences, goal attainment, and future maintenance of acquired skills; and v) a ‘booster’ day-long workshop 3 months post-surgery. The rationale for this ‘booster’ session is based on a prior randomised trial in which we found that improvements in pain and physical function after TJA plateau by 6 months. Patients with sub-optimal improvements in pain and physical function at 6 months demonstrated a significant decline in their mental well-being scores at 12 months compared to those who experienced optimal physical function, whose mental well-being scores were maintained [29].

### Standard of care

Patients will undergo surgery and post-operative care as per SVHM’s routine TJA program which has been standardised through the use of clinical pathway protocols and validated in a randomised controlled trial [30]. Post-discharge rehabilitation involves either an in-patient or home-based physiotherapy program which is pre-determined during pre-admission clinic assessment using a validated discharge predictor tool [31]. Both programs are standardised and conducted through SVHM’s in-patient or Hospital In-The-Home service and are followed by referral to a community-based physiotherapy program, based on the locality of the patient’s residence. At a pre-admission clinic, all patients receive an instruction booklet outlining the continuum of care for TJA. All patients are reviewed post-surgery in the same clinic at intervals of 6-weeks, 3-, 6-, and 12-months, and annually thereafter.

### Outcome assessments

#### Data capture

Outcomes will be captured by the research associate. Demographic information collected at baseline will include age, gender, co-morbidities, body mass index, and socioeconomic data. We will also collect ‘time’ on the waiting list. All MT sessions will be audio-taped and a random sample will be selected and assessed for adherence to the principles of MT. Fully-itemised costs of healthcare will be captured via SVHM’s hospital network, including administered medications as well as costs of hospital in-patient and out-patient encounters. While beyond the scope of the initial Discovery Project time frame, the investigators will include a longer term follow-up plan in the ethics application to allow scope for continued data capture in the study group at the annual outpatient review to 10 years.

#### Primary outcome

Will be self-reported pain and physical function measured on the Western Ontario and McMaster Universities (WOMAC) Osteoarthritis Index [32]. The WOMAC consists of 24 items covering three subscales: pain (five items), stiffness (two items), and physical function (17 items). The pain and physical function subscales will be used, each subscale transformed to a score ranging from 0 to 100, with a higher score indicating greater pain and physical function. The WOMAC is a widely-used questionnaire specifically designed to evaluate knee and hip osteoarthritis [33,34].

#### Secondary outcomes

● Psychological well-being: will be derived from the 12-item Short Form Health Survey mental component score [27].

● Quality of Life: will be measured using the WHOQOL-BREF, a 26-item quality of life (QoL) Assessment [35].

● Depression and anxiety: data will be derived from the Hospital Anxiety and Depression Scale (HADS), a validated instrument for detecting states of depression and anxiety and severity of emotional disorder in patients under treatment in hospital medical, surgical, and outpatient departments [36].

● Self-efficacy: will be measured using the Arthritis Self-Efficacy Scale, a 20-item questionnaire [37].

● Mindfulness: the Mindful Awareness Attention Scale (MAAS) is a 15-item measure assessing mindfulness and its role in psychological well-being [38].

● Carver brief COPE questionnaire: a 28-item questionnaire to measure coping styles [39].

● Brief illness perceptions questionnaire: a 9-item measure of illness perceptions [40].

● Irrational scale: a 20-item measure of cognitive distortions [41].

● Health services utilisation: information regarding utilisation of health services, including medications and in- and outpatient services, will be captured by linkage of our database to administrative and clinical databases maintained by SVHM. The latter capture extensive information on all healthcare encounters (in-patient and out-patient) occurring at SVHM’s hospital network, including administered medications as well as fully-itemised costs of healthcare encounters.

#### Additional measures

● The Mini-Mental State Exam (MMSE) [42]. This short test is a commonly used screening instrument of cognitive function with scores ranging from 0 to 30. MMSE scores lower than 24 are reliably associated with the diagnosis of dementia or other organic mental disorders. Although patients with frank dementia will have been detected and excluded from the study, it is possible that, in this patient population, some subjects will have previously unrecognised mild degrees of cognitive impairment, and thus that cognition may act as a confounder.

● Charlson Comorbidity Score [43]. This is a weighted index for classifying comorbidities’ severity, validated for estimating the risk of morbidity and mortality in longitudinal studies.

● Self-reported past psychiatric history will be obtained during the pre-surgery assessment to account for previous psychological problems.

● Participants will be asked to rate their adherence to mindfulness practice at 12 months through a questionnaire that includes a Likert scale assessing individual motivation to continue mindfulness practice and questions regarding the average amount of time (minutes) spent engaging in formal and informal meditation.

### Timelines

**Figure 1:**
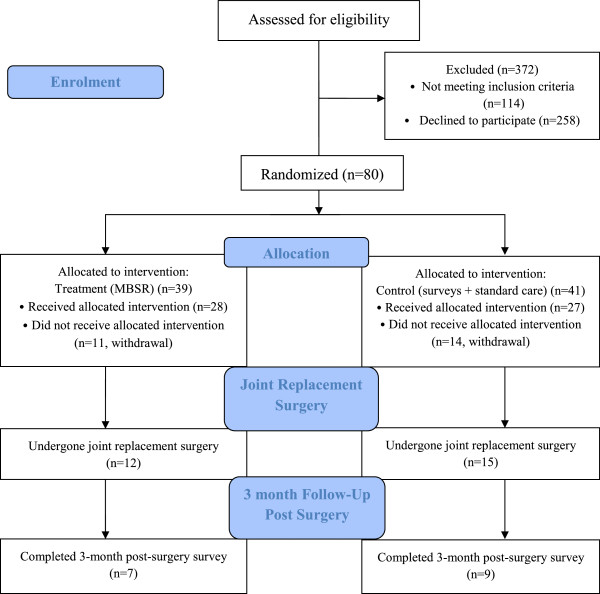
Trial flow diagram–first 12 months of study.

This is a 3-year study commencing June 2012 and ending May 2015. See Table 1 and Figure 1 for time points and recruitment progress.

**Table 1:** Trial study visits

**Documentation**	**Visit 1**	**Visit 2**	**Visits 3–11**	**Visit 12**	**Visit 13**	**Visit 14**
Baseline data, demographics	X					
Eligibility criteria	X					
Randomisation	X					
Baseline assessments*		X				
8-week MT program+			X			
Total joint arthroplasty				X		
3 month MT booster session+					X	
3 month assessments*					X	
12 month assessments*						X

*Assessments are given to patients at their surgical review visit but completed independently. Assessments include: WOMAC; SF-12; WHOQOL-BREF; HADS; Arthritis Self-Efficacy Scale; MAAS, Carver Brief COPE; Brief Illness Perceptions Questionnaire; MMSE (administered at baseline during the pre-admission assessment).

+Applies to Attendance for Intervention Group Only.

### Sample size

The sample size calculation was based on the following parameters: i) alpha value = 0.05, 2-sided; ii) power = 80%; iii) expected rates of the primary outcome 1-year post-TJA. To determine the sample size, we reviewed the data from our SVHM joint registry from 2009 to 2010 [44]. Of 1,180 patients who underwent TJA, 40% showed moderate to severe pre-operative psychological distress according to the pre-operative SF-12 MCS score. The mean difference in functional outcome scores at 12 months post-TJA between individuals with moderate to severe pre-operative psychological distress and individuals with no or mild psychological distress was 10 (+20) points. Based on these data, the sample size required in each of the 2 groups is 64. To allow for 15% attrition, 150 patients will be recruited in total. These numbers will also give more than 80% power to detect the expected minimum clinically important difference in pain and functional outcome scores between the combined MT + TJA group and the TJA alone group.

### Recruitment

Based on data from our registry, if we assume 40% of patients will present with moderate to severe psychological distress and exclude patients who have a poor command of English (10%), 450 patients would be eligible for inclusion over a 20-month recruitment period. Presuming a conservative recruitment rate of 1 in 3, target recruitment is achievable within the study timeframe. Potential participants will receive both verbal and written information about the requirements of participation including i) verbal explanation of the program content upon initial contact and ii) detailed outline of the program content and required commitment in the patient information and consent form. Consistent with our existing recruitment strategy for randomised controlled trials, patients will receive i) written material for study participants and ii) travel assistance and telephone reminders of scheduled appointments; strategies proven to improve recruitment and retention rates in interventional studies [45]. Of importance and to avoid bias, these strategies will be employed with patients in both study arms.

### Randomisation and masking

Following consent, block randomisation will be used to determine which participant receives the intervention according to a computer-generated random assignment sequence prepared in advance. Considerable effort will be made to avoid observer bias through separation of roles and blinding of trial staff. A research assistant independent of patient recruitment and data collection will be responsible for co-ordination of patient appointments, telephone reminders, and travel assistance. The research associate (who will be responsible for patient consent) will be blinded to group allocation. Mindfulness training will be conducted by investigators who will remain blinded to outcome data throughout the study. Upon completion of the study, a biostatistician blinded to group allocation will analyse outcome data.

### Data management

All data is stored on a password protected computer kept in a secure locked facility and only accessible to the Chief Investigators and Trial’s Coordinator as approved by the SVHM Human Research Ethics Committee. At the completion of the study, outcome data will be pooled and de-identified for analysis by a statistician.

### Statistical analysis

Statistical analysis will be by intention to treat. Categorical variables will be analysed using χ^2^ tests (or Fisher’s Exact test for small samples) while for continuous variables we will employ (parametric) *t*-tests and (non-parametric) Mann-Whitney tests for symmetrically and asymmetrically distributed data, respectively. The significance of differences in dichotomous data will be tested using generalised estimating equations or linear mixed model. If there are chance imbalances in baseline patient characteristics hypothesised to influence the main outcomes, then statistical techniques that allow adjustment for confounding variables will be used as secondary analyses. If there are more than 5% missing data, sensitivity analyses allowing for different assumptions, such as the best or worst possible scenario, will also be reported for the main outcomes of the study.

Health economic modelling will be applied to estimate the potential cost-effectiveness of MT + TJA. Decision analysis [46] will be used to compare the downstream consequences of MT + TJA versus TJA alone. The incorporation of Markov [47] and life-tabling [48] techniques will allow for the modelling of outcomes beyond one year. The main output of interest in health economic modelling is incremental cost-effectiveness ratios in terms of net costs per unit of health gain. Net costs will comprise the costs of MT + TJA minus costs saved from the reduction in downstream health services utilization. Health gains will be measured by estimating years of life gained and quality-adjusted life years gained. Both are enabled by the collection of time-to-outcome data and the latter also by collection of QoL data. All health economic analyses will be undertaken in accordance with recommended approaches, such as 5% discounting of estimated future costs and health gains. To account for any uncertainty in the data inputs for health economic modelling, sensitivity and uncertainty analyses will be undertaken via Monte Carlo simulation [49].

## Discussion

Experimental research on the ‘mechanism of action’ of MT is still evolving, but there are good theoretical reasons to suggest MT will be effective in this patient group. Our clinical experience shows that despite much education and informed consent, many patients have exaggerated, unrealistic expectations of TJA, leading to greater postoperative dissatisfaction [50]. This often goes with a more passive role in their own recovery, lower self-efficacy, greater reliance on pain medication, and worse outcomes generally, all often aggravated by having to wait long periods for the procedure. As MT teaches patients to come to terms with unpleasant experiences without becoming caught up in mental elaborations (e.g., disappointment or anger), which accentuate the initial, unavoidable kernel of suffering [25], it is likely to enhance acceptance of partial symptomatic recovery. Physical rehabilitation is likely to benefit from the greater awareness of body sensations and limitations, as well as the confidence gained in exploring those limits, encouraged by a number of MT methods (notably ‘body scan’ and yoga) [51]. MT helps patients come to terms with the pain as it is, rather than employing distraction and pain reduction strategies which increasingly are recognised to impair psychological function when pain is chronic [52].

As already suggested, there is an extensive direct evidence base for efficacy of MT in related conditions, such as chronic pain [53]. Some of the earliest trials of MT involved chronic pain patients, in whom it improved coping and adaptation and reduced maladaptive illness behaviours such as inappropriate health system utilisation [54]. Effect sizes in studies of MT in general hospital settings compare favourably with other psychological interventions; for example, meta-analysis of studies in cancer patients found an effect size (relative risk) of 0.48 for psychological and 0.18 for physical outcomes [55]. While broad in its application and associated with accruing evidence for long-term functional and quality of life benefits [21], there are as yet no harmful side effects reported in MT.

While MT has been shown in numerous intervention studies to enhance psychological and physiological well-being in chronic disease populations [53], possible mechanisms or mediators of these health outcomes have been less well investigated [56]. In particular, little research has addressed the connections between mindfulness and well-established concepts in health psychology, such as illness beliefs, cognitive distortions, and coping styles. A large body of research shows that certain perceptions of illness (e.g., perceived controllability) and cognitive distortions (e.g., “life should be easier”, “to be worthwhile I must do a thoroughly competent job in everything I do”) are associated with greater symptom reporting and reduced activity [57], greater anxiety and depression [56,58,59], and reduced QoL [60]. Coping styles, that is, the ways in which individuals try to manage challenges, have been identified as the key mediator of the relationship between illness perceptions, illness severity, and illness outcomes (such as QoL, depression, and anxiety) [57,61]. While the literature on coping is complex, a key distinction is between ‘approach’ coping, generally associated with improved health outcomes, and ‘avoidant’ coping, typically associated with poorer health outcomes [56].

To date, only one published study has specifically evaluated the influence of MT on coping styles and possible mediating cognitive processes [56]. Using a sample of 57 students, the authors found that after 12 weeks of mindfulness classes, cognitive distortions, approach coping, and positive affect increased, while avoidance coping and anxiety and negative affect decreased. Encouraging as these results may be, their applicability to an older, physically unwell, and psychologically troubled population remains an open question.

Given the efficacy of MT, the lack of information about its effects on well-established mediators of illness outcomes is an important gap in the literature. Thus, an important research question is the extent to which the beneficial impact of MT on illness outcomes may be due to changes in individual illness perceptions, cognitive distortions, and coping styles. Our exploratory investigation of this question will help guide future mindfulness-based interventions and research.

This study will use a mindfulness-based psychological intervention to enhance outcomes in people undergoing total joint replacement and, in so doing, will test hypotheses about coping with chronic illness in an aged population.

### Ethical considerations

This study was approved by the St. Vincent’s Hospital Human Research Ethics–(HREC-A 143/11) and was registered on the Australian New Zealand Clinical Trials Registry (ANZCTRN12611001184965) 15th November 2011. Written informed consent is obtained by the Trials coordinator from all study participants prior to enrolment. Patients are free to withdraw from the trial at any time without providing a reason. Details outlining participant rights to access to their data, study results, how study data will be used, and the processes in place to ensure individual confidentiality is protected are all specified in the participant information and consent form.

## Trial status

Open to recruitment.

## Abbreviations

HADS: Hospital Anxiety and Depression Scale; MAAS: Mindful Awareness Attention Scale; MCQ: Memory Complaints Questionnaire; MMSE: Mini-Mental State Exam; MT: Mindfulness-based stress reduction; QoL: Quality of life; SF-12: Short form-12; SVHM: St. Vincent’s Hospital Melbourne; TJA: Total joint arthroplasty.

## Competing interests

The authors declare that they have no competing interests.

## Authors’ contributions

PC is the lead principal investigator of the study, has full access to all of the data in the study, and takes full responsibility for the integrity of the data and for the accuracy of the data analysis. None of the authors have financial or personal interest affiliations with the sponsors of this research effort. All authors (MD, DC, SK, KM, MS, PC) developed the design and study protocol, the funding application, and ethics submission. MD drafted the manuscript and MS, SK, DC, and PC critically revised the manuscript. All authors read and approved the final version.
